# Early-Onset Schizophrenia With Predominantly Negative Symptoms: A Case Study of a Drug-Naive Female Patient Treated With Cariprazine

**DOI:** 10.3389/fphar.2020.00477

**Published:** 2020-04-23

**Authors:** Maria Judit Molnar, Idris János Jimoh, Helga Zeke, Ágnes Palásti, Marianna Fedor

**Affiliations:** Institute of Genomic Medicine and Rare Disorders, Semmelweis University, Budapest, Hungary

**Keywords:** cariprazine, schizophrenia, negative symptoms, early-onset schizophrenia, second-generation antipsychotic

## Abstract

Schizophrenia is a chronic and severe mental disorder characterized by positive, negative, and cognitive symptoms. Negative symptoms are usually present from the prodromal phase; early diagnosis and management of negative symptoms is a major health concern since an insidious onset dominated by negative symptoms is associated with a worse outcome. Antipsychotic medications, which are effective for treating positive symptoms, are generally ineffective for treating negative or cognitive symptoms. We present a 23-year-old woman showing severe symptoms at her first visit to our department. The patient’s parents reported that their daughter had experienced several years of psychosocial decline and putative psychiatric symptoms, but no medical attention had been previously sought; as such, the diagnosis of schizophrenia with predominantly negative symptoms was very much delayed. Early onset of schizophrenia, longer duration of untreated psychosis, and severe negative symptoms, which have limited treatment options, suggested a poor prognosis. We initiated monotherapy with cariprazine, a novel antipsychotic that has recently been proven efficacious in treating schizophrenia with predominantly negative symptoms. This report describes a 52-week cariprazine treatment regimen and follows the patient’s impressive clinical improvement confirmed by PANSS and CGI scores, and psychological tests.

## Background

Schizophrenia is a severe, chronic, and heterogeneous mental disorder that often has debilitating long-term outcomes. Its lifetime prevalence rate is estimated to be approximately 1% worldwide in the adult population (Lehman et al., 2010). Onset generally occurs in late adolescence or early adulthood, with an average age of 18 years for men and 25 years for women.^1^ The term early-onset schizophrenia (EOS) is used to refer to patients who are diagnosed with the disorder before this age. EOS is a severe, frequently disabling, and chronic condition with a prevalence approaching 0.5% in those younger than 18 years (Hafner and Van der Heiden, 1997).

Schizophrenia is accompanied by a distortion of personality that affects fundamental mental and social functions, making everyday life extremely difficult for patients. Clinical symptoms are often classified in three main domains: positive symptoms, such as hallucinations, delusions, suspiciousness/persecution; negative symptoms, such as emotional withdrawal, blunted affect, and passive social withdrawal; and cognitive symptoms, such as impaired perception, learning, thinking, and memorizing. EOS may be accompanied by greater symptom severity, premorbid developmental impairment, ‘soft’ neurological signs (eg, clumsiness, motor incoordination), and a higher rate of substance abuse (Hsiao and McClellan, 2008; Clemmensen et al., 2012; Immonen et al., 2017). Accordingly, diagnosis of EOS is often difficult and frequently delayed since onset is more commonly insidious than acute, which makes it difficult to differentiate EOS from underlying cognitive deficits, premorbid functional impairment, or other abnormalities (Russell, 1994; Bartlet, 2014). Given this common delay in recognition of the disorder, the duration of untreated psychosis is often very long, further contributing to a poor outcome (Penttila et al., 2014).

Although various hypotheses have been developed, the etiopathogenesis of schizophrenia and EOS is not fully understood (McGuffin, 2004; Klosterkotter et al., 2011).^2^ Among the rising and falling neurochemical theories, the dopamine hypothesis has remained a primary hypothesis guiding the treatment of schizophrenia. There are four dopaminergic pathways in the human brain: the mesolimbic, the mesocortical, the tuberoinfundibular, and the nigrostriatal. Positive symptoms of schizophrenia are associated with the hyperdopaminergic state of D_2_ receptors in the mesolimbic area, while negative and cognitive symptoms are believed to be related to the hypodopaminergic dysregulation of the prefrontal cortex (Stahl, 2003).

Negative symptoms of schizophrenia, which affect up to 60% of patients with schizophrenia (Rabinowitz et al., 2013), form a complex clinical constellation of symptoms that challenge both diagnosis and treatment. By definition, negative symptoms mean the absence of normal functions. Negative symptoms are classified by their etiology as primary negative symptoms, which are core features of the disease itself, and secondary negative symptoms, which are consequences of positive symptoms, antipsychotic treatment, depression or extrapyramidal side effects. Five constructs have been accepted by general consensus as key aspects of negative symptoms: blunted affect, alogia, anhedonia, asociality, and avolition (Marder and Galderisi, 2017). Patients with predominant negative symptoms lose their motivation, cannot function at school or work, and their interpersonal relationships severely decay. Due to impaired daily functioning and social amotivation, they may need constant care.

Although early intervention is associated with improvement in negative symptoms (Boonstra et al., 2012), this may be challenging since negative symptoms develop slowly and may be difficult to detect or differentiate from other clinical features (Kirkpatrick et al., 2001; Galderisi et al., 2018). Moreover, a more insidious onset predicts poorer outcome and more severe negative symptoms (Kao and Liu, 2010; Immonen et al., 2017; Murru and Carpiniello, 2018). Diagnosis of patients with predominantly negative symptoms (lacking manifest psychotic signs) is often delayed, resulting in a longer duration of untreated psychosis. The length of untreated psychosis is closely related to poorer functional outcome (Perkins et al., 2005).

Negative symptoms have traditionally had minimal response to antipsychotic treatment. First-generation antipsychotics are effective in treating positive symptoms, but negative symptom improvement is only evident when symptoms are secondary to positive symptoms. It was initially hoped that second-generation antipsychotics would target both positive and negative symptoms, but efficacy data have been disappointing. This was a large meta-analysis where only four second-generation drugs (amisulpride, risperidone, olanzapine, and clozapine) resulted to be more efficacious than first-generation antipsychotics in the overall change of symptoms, including positive and negative symptoms. The other examined second-generation antipsychotics were only as efficacious as first-generation antipsychotic agents (Leucht et al., 2009). These studies were mainly conducted in patients with general symptoms of schizophrenia, therefore a secondary effect on negative symptoms could not be ruled out. Therefore negative symptom improvement cannot be considered a core component of atypicality (Veerman et al., 2017). Previous studies have demonstrated that no drug had a beneficial effect on negative symptoms when compared to another drug (Arango et al., 2013; Millan et al., 2014; Fusar-Poli et al., 2015), meaning that head to head comparisons of different agents among each other did not result in superiority of one drug to another. The latest comparison (Krause et al., 2018) evaluated all studies that have been performed in the negative symptom population so far, and has found that amisulpride claimed superiority only to placebo, olanzapine was superior to haloperidol, but only in a small trial (n = 35), and cariprazine outperformed risperidone in a large well-controlled trial.

Hence cariprazine emerged as an agent of particular interest in regard to negative symptoms. Cariprazine is a dopamine D_3_/D_2_ receptor partial agonist and serotonin 5-HT_1A_ receptor partial agonist. It has been hypothesized that cariprazine is the only antipsychotic that can block D_3_ receptors in the living brain, thereby exhibiting functions that are related to D_3_ blockade (e.g., improvement of negative symptoms) (Stahl, 2016). In that large clinical trial including 460 patients with predominant negative symptoms and stable positive symptoms of schizophrenia, cariprazine was significantly more effective than risperidone in improving negative symptoms and patient functioning (Nemeth et al., 2017).

## Case Description

The 23-year-old female patient visited the Institute of Rare Diseases at our university with her parents. They had suspected for a long time that something was wrong with their daughter, but this was the first time they had asked for medical help. The patient was quiet and restrained since she did not speak much, her parents told us her story instead. Initially, the patient had done very well in a bilingual secondary school and was socially active with friends and peers. At the age of 15 years, her academic performance started to deteriorate, with her first problems associated with difficulty learning languages and memorizing. Her school grades dropped, and her personality started to gradually change. She became increasingly irritated, and was verbally and physically hostile toward her classmates, resorting to hitting and kicking at times. She was required to repeat a school year and subsequently dropped out of school at the age of 18 because she was unable to complete her studies. During these years, her social activity greatly diminished. She lived at home with her parents, did not go out with friends, or participate in relationships. Most of the time she was silent and unsociable, but occasionally she had fits of laughter without reason. Once the patient told her mother that she could hear the thoughts of others and was probably hearing voices as well. Slowly, her impulse-control problems faded; however, restlessness of the legs was quite often present.

Our patient’s medical history was generally unremarkable. She lacked neurological or psychiatric signs. She had a tonsillectomy and adenotomy at age 7 years. Epilepsy was identified in the patient’s family history (father’s uncle). On physical examination, there were no signs of internal or neurological disease; body mass index was 21.5 (normal weight).

During the first psychiatric interview and examination, we found that our patient was alert and vigilant, but had trouble relating due to decreased integrity of consciousness. Her attention could be aroused or partially directed, and she had difficulty keeping a target idea. Autopsychic and allopsychic orientations were preserved. Longer thinking latencies and slowed movement responses were observed, sometimes with even cataleptic impressions. Cognitive functions, such as thinking, memory, and concept formation, were severely impaired, and we were unable to carry out some of our neurocognitive tests -such as the Addenbrooke’s Cognitive Examination (Hsieh et al., 2013), the Toulouse-Pieron attention test (Kanizsa G1951), Bells test (Gauthier et al., 1989) and the Trail Making Test- because of the patient’s denial of symptoms and refusal to cooperate.

She often looked aside and laughed frequently, suggesting the presence of perceptual disturbances, but she denied her symptoms when asked. In contrast to the periodic inappropriate laughing, apathy and anhedonia were markedly present. During the examination, the patient could not recall anything she would do or even think of with pleasure. According to the heteroanamnesis, she lost her interest in activities she used to like, did not go out with friends anymore, and showed no signs of joy or intimacy towards her family members either.

Along with the affective hyporesponsiveness, amotivation and a general psychomotor slowing were observed. Hypobulia, void perspectives, and lack of motivation were explored. Parental statements indicated that the patient’s social activity had continued to diminish, and her appearance and personal physical hygiene had deteriorated. When we initiated a conversation, the patient was negativistic and agitated. Her critical thinking ability was reduced, which led to inappropriate behavior (she, e.g., unexpectedly stood up and left the room while the examination was still ongoing). Considering her status, she was admitted to the clinic after her first visit.

After several differential diagnostic tests were performed (e.g., routine diagnostic laboratory parameters, immune serological analyses, electroencephalogram, magnetic resonance imaging, genetic testing), all the possible common and rare disorders, such as Huntington’s disease, Niemann Pick C disease, mitochondrial disorders, and autoimmune diseases, were ruled out.

At first contact, to differentiate the symptoms and severity of putative schizophrenia, we mapped the positive, negative, and general symptoms, as well as a clinical impression, using the Positive and Negative Syndrome Scale (PANSS), the Scale for Assessment of Negative Symptoms, and the Clinical Global Impressions-Severity (CGI-S) (Groth-Marnat, 2009).

The patient had a very high PANSS total score, which corresponded to being considered “severely ill” or “among the most severely ill’ on the CGI-S (Leucht et al., 2005). The PANSS score was derived dominantly from the negative items of the scale. Overall, her negative symptoms fulfilled criteria for predominantly negative symptoms, meaning that positive symptomatology was reduced, while negative symptoms were more explicit and dominated the clinical picture (Riedel et al., 2005; Olie et al., 2006; Mucci et al., 2017). Baseline rating scale sores are presented in **Table 1**.

**Table 1:** Summary of symptom scale scores at the time of admission to the hospital.

	Score at first contact/admission to hospital	Maximum score possible
PANSS Total Score	150	210
PANSS Positive Subscale Score	26	49
PANSS Negative Subscale Score	45	49
PANSS General Psychopathology Subscale Score	79	112
PANSS-FSPS	20	42
PANSS-FSNS	46	49
CGI-S	6	7

PANSS, Positive and Negative Syndrome Scale; PANSS-FSNS, Positive and Negative Syndrome Scale factor score for negative symptoms; PANSS-FSPS, Positive and Negative Syndrome Scale factor score for positive symptoms; CGI-S, Clinical Global Impressions-Severity Scale.

The diagnosis of EOS with predominantly negative symptoms was given and treatment with the antipsychotic agent cariprazine was initiated. The patient was hospitalized for 2 weeks following her arrival at the clinic. Cariprazine was started at the dose of 1.5 mg/day and titrated up to 4.5 mg/day over a 2-week period: the patient received 1.5 mg/day for the first 3 days, 3 mg/day from day 4 to day 12, and eventually 4.5 mg/day from day 13 onward. During these 2 weeks, which were spent in hospital, the patient’s explicit negative symptoms such as poverty of speech, psychomotor retardation, poor eye contact, and affective nonresponsiveness improved; however, delusions and hallucinatory perceptions did not fade significantly.

Two weeks after discharge, we saw the patient for her first outpatient visit. Significant clinical improvement was observed. The patient calmly cooperated during the examination, with no signs of agitation. She was oriented to time, place, and self, attention could be drawn and directed, and she was able to keep a target idea and change the subject. Although according to the family, perceptual disturbances were still present, laughing with no reason and looking aside were much less frequent, and restlessness of the legs had stopped; these symptoms were not observed during the examination. Psychomotoric negativism had improved greatly, the patient was more communicative, and she paid more attention to the activities of family members. The pace of speech was close to normal: the thinking latencies and slowed movement responses as observed at admission were not seen anymore. The patient had adequate reaction time to questions asked and could focus in the interview. Mild obstipation and somnolence in the evening were her main complaints. Apart from some tick-like eye closures, there was no pathological finding during physical and neurological examination. At this point, cariprazine was reduced to 3 mg per day.

At her second outpatient visit, which occurred 8 weeks after treatment initiation, further improvement was observed. According to her mother, the patient was more active and open at home. Neurological examination found that the alternating movements of her fingers were slightly slowed. Cariprazine 3 mg/day was continued with concomitant anticholinergic medication.

At the third outpatient visit, which occurred 16 weeks after the first contact, the patient’s overall symptoms, including cognitive functions, such as memory and abstract thinking, as well as functions in activities of daily living, had improved remarkably. She had started to participate in the family’s daily life, even taking responsibility for some household duties; further, she went to the hairdresser for the first time in years, a step forward from her previous state of self-neglect. She was probably still having auditory hallucinations, which she considered natural, and some extrapyramidal symptom (EPS)-like ruminating movements, like to-and-fro swinging of her trunk, were observed. She did not look aside any more and tics were no longer present. Compared with previous visits overall, she was very relaxed, retained eye contact, cooperated, and communicated adequately during the interview. She started to develop insight into her condition, and she told us that her “thoughts were not healthy.” At the last two visits, the synkinesis of the arms was reduced.

After 16 weeks of treatment, the patient’s PANSS Negative Subscale Score and PANSS factor score for negative symptoms (PANSS-FSNS) score were reduced by 44.44% and 41.31%, respectively. Recent studies have demonstrated that linking the percentage improvement of PANSS with CGI-S and -Improvement (CGI-I) scores shows that a 25–50% reduction of PANSS scores corresponds to clinically meaningful change (Correll et al., 2011; Fusar-Poli et al., 2015). In acutely ill patients with predominantly positive symptoms who are more likely to respond well to treatment, the 50% cutoff would be a more clinically meaningful criterion; however, since even slight improvement might represent a clinically significant effect in a patient with atypical schizophrenia, the use of 25% cutoff is justified (Correll et al., 2011; Fusar-Poli et al., 2015).

In this regard, the 44.44% (change from baseline: −20) and 41.31% (change from baseline: −19) improvement demonstrated on PANSS Negative Symptom subscale and PANSS-FSNS, respectively, are considered a clearly clinically relevant change. Beyond the impaired synkinesis and alternating movement of the arms and fingers, there were no other treatment-related physical dysfunctions. Change from baseline on the PANSS and CGI scales are shown over the course of treatment in **Table 2**.

**Table 2:** Summary of symptom scale scores at weeks 16, 32, and 52.

	Score at admission	Score at 16 weeks of treatment	Percent change from baseline (change from baseline)	Score at 32 weeks of treatment	Percent change from baseline (change from baseline)	Score at 52 weeks of treatment	Percent change from baseline (change from baseline)
PANSS Total Score	150	92	38.66% (−58)	37	75.33% (−113)	37	75.33% (−113)
PANSS- PSS	26	14	46.15% (−12)	9	65.38% (−17)	9	65.38% (−17)
PANSS- NSS	45	25	44.44% (−20)	15	66.67% (−30)	15	66.67% (−30)
PANSS-FSPS	20	9	55.00% (−11)	6	70.00% (−14)	6	70.00% (−14)
PANSS-FSNS	46	27	41.31% (−19)	14	69.56% (−32)	14	69.56% (−32)
CGI-S	6	4	NA	2	NA	2	NA
CGI-I	NA	2	NA	1	NA	1	NA

PANSS, Positive and Negative Syndrome Scale; PANSS-PSS, PANSS Positive Subscale Score; PANSS-NSS, PANSS Negative Subscale Score; PANSS-FSNS, Positive and Negative Syndrome Scale factor score for negative symptoms; PANSS-FSPS, Positive and Negative Syndrome Scale factor score for positive symptoms; CGI-S, Clinical Global Impressions-Severity Scale; CGI-I, Clinical Global Impressions-Improvement Scale. NA indicates not applicable.

Since our patient’s symptoms demonstrated strong improvement and tolerability was favorable, cariprazine therapy was continued. Improvement in both negative and positive symptoms was maintained over the course of treatment. At her later visits (32 and 52 weeks), PANSS total score was reduced to a level that was close to the minimum, and the decrease in negative symptom scores was considerable (PANSS-NSS=66.67% and PANS-FSNS=70.00% at both time points). The patient’s progress was also reflected in clinical and functional measurements, with the CGI-S score reduced to 2 (borderline mentally ill) and a CGI-I score of 1 (very much improved) indicating notable improvement.

## Discussion

Cariprazine has demonstrated broad spectrum efficacy in the treatment of positive and negative symptoms of schizophrenia. In a field where no treatment is available for difficult-to-treat negative symptoms, this case is unique and may have important implications for schizophrenia treatment. Despite experiencing approximately 8 years of untreated symptoms and functional impairment associated with predominantly negative symptom EOS, our 23-year-old female patient showed considerable symptomatic and functional improvement after several weeks of treatment with cariprazine. Given that the duration of untreated negative symptoms is associated with worse functional outcomes (Boonstra et al., 2012), the remarkable improvement seen in this case shows how valuable cariprazine could be for patients with similar symptom presentations. Although it is not possible to generalize the observations and findings of this single case, it has the novelty of detecting a potential effect of cariprazine in a drug-naïve patient with marked negative symptoms of early-onset schizophrenia. To our knowledge, no cariprazine-related data has been published in this type of patients. A single case study is obviously far from being predictive for the efficacy of a drug, however, the results seen with this case are promising. With a dose recommended for patients with negative symptoms, our patient’s clinical condition, including positive, negative, and cognitive symptoms, as well as social functioning have improved notably, with the effect maintained for over 12 months. Generally, cariprazine has been well tolerated, with mild EPS observed after 8 weeks, but no metabolic, cardiac, or other side effects.

## Conclusion

This case report suggests that the management of patients with EOS and prominent negative symptoms is achievable in everyday practice with cariprazine. More real-world clinical experience is needed to support this finding.

## Data Availability Statement

All datasets generated for this study are included in the article/supplementary material.

## Ethics Statement

Written informed consent was obtained from the individual(s), and minor(s)’ legal guardian/next of kin, for the publication of any potentially identifiable images or data included in this article.

## Author Contributions

All authors listed have made substantial, direct, and intellectual contribution to the work and approved it for publication.

## Funding

This work was supported from Research and Technology Innovation Fund by the Hungarian National Brain Research Program (KTIA_NAP_ 2017-1.2.1-NKP-2017-00002). Editorial support for this case report was supported by funding from Gedeon Richter. The funder was not involved in the study design, collection, analysis, interpretation of data, the writing of this article or the decision to submit it for publication.

## Conflict of Interest

The authors declare that the research was conducted in the absence of any commercial or financial relationships that could be constructed as a potential conflict of interest.

## Footnotes

1
http://www.schizophrenia.com/szfacts.htm

2
https://www.nimh.nih.gov/health/topics/schizophrenia/index.shtml
